# A novel case of aripiprazole and clomipramine-induced toxic megacolon: enough to drive anyone mad

**DOI:** 10.1093/jscr/rjaf265

**Published:** 2025-06-23

**Authors:** Taylor J Casey, Britton P Mehr

**Affiliations:** Department of General Surgery, UPMC Harrisburg, 111 S Front Street, Harrisburg, PA 17101, United States; Department of General Surgery, UPMC Harrisburg, 111 S Front Street, Harrisburg, PA 17101, United States

**Keywords:** toxic megacolon, antipsychotics, chronic constipation

## Abstract

Toxic megacolon is a potentially lethal disease process in which there is a non-mechanical dilation of the colon to >6 cm, often associated with systemic effects including leukocytosis or hemodynamic compromise. Here, we present a case of a 49-year-old male who presented with idiopathic toxic megacolon who underwent a subtotal colectomy with end ileostomy. Pathology confirmed no inflammatory bowel disease or *Clostridium difficile* infection, the most common etiologies. This was a unique case in that the patient did not present with the most common etiology, but did have a history of chronic constipation in the setting of antipsychotic/antidepressant use. Scattered cases of antipsychotic-induced toxic megacolon have been described in the literature, but to the author’s knowledge, none related to aripiprazole or clomipramine. This case highlights the importance of diligent clinical monitoring for patients on psychiatric medications.

## Introduction

Toxic megacolon (TM) is a potentially lethal condition caused by severe, non-obstructive dilation of the colon >6 cm, associated with systemic signs such as pyrexia, tachycardia, hypotension, and/or leukocytosis [1, 2]. Historically, it has been most commonly linked to inflammatory bowel disease, with prior studies being inconclusive regarding whether Crohn’s disease or ulcerative colitis made the patient higher risk [3]. However, there has been a rise in infectious etiologies in the last few years given advancements in medical management of these diseases, most commonly *Clostridium difficile* in the general population and cytomegalovirus in the immunocompromised [4].

The pathogenesis of TM has not been entirely elucidated, but is believed to be related to inflammatory mediator release and induction of smooth muscle relaxation [5]. Release of nitric oxide synthase induces further smooth muscle relaxation and dilation, causing colonic dysmotility. After migration to the site due to the inflammatory cascade, neutrophils cause further damage by the release of cytokines, proteolytic enzymes, and leukotrienes, which also cause colonic dilation [6]. Once the diagnosis is made, urgent initiation of complete bowel rest, intravenous hydration, and broad-spectrum antibiotics is required. If initial conservative measures fail, surgical intervention is required, with total colectomy and end ileostomy being the procedure of choice [7].

Here, we present a case of a 49-year-old male who was prescribed the antipsychotic medications aripiprazole, clomipramine, and venlafaxine who presented with TM of unknown etiology. Tissue pathologic analysis confirmed the presence of diffuse ischemia in the absence of inflammatory bowel disease as well as lack of thrombi in the vessels.

## Case report

**Figure 1:**
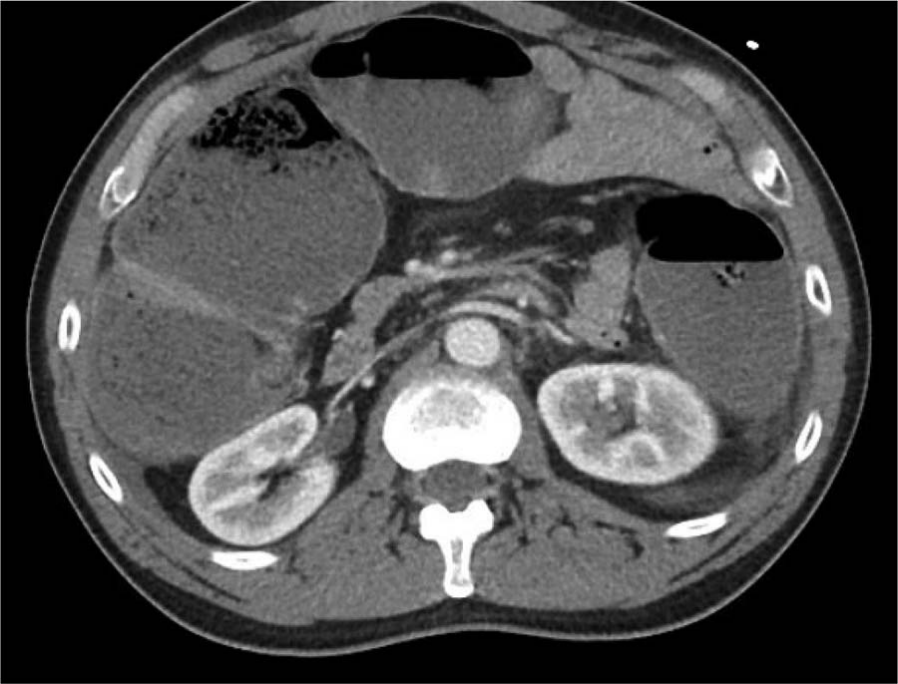
Axial imaging demonstrating significant colonic dilation. There was no evidence of pneumatosis or pneumoperitoneum.

**Figure 2:**
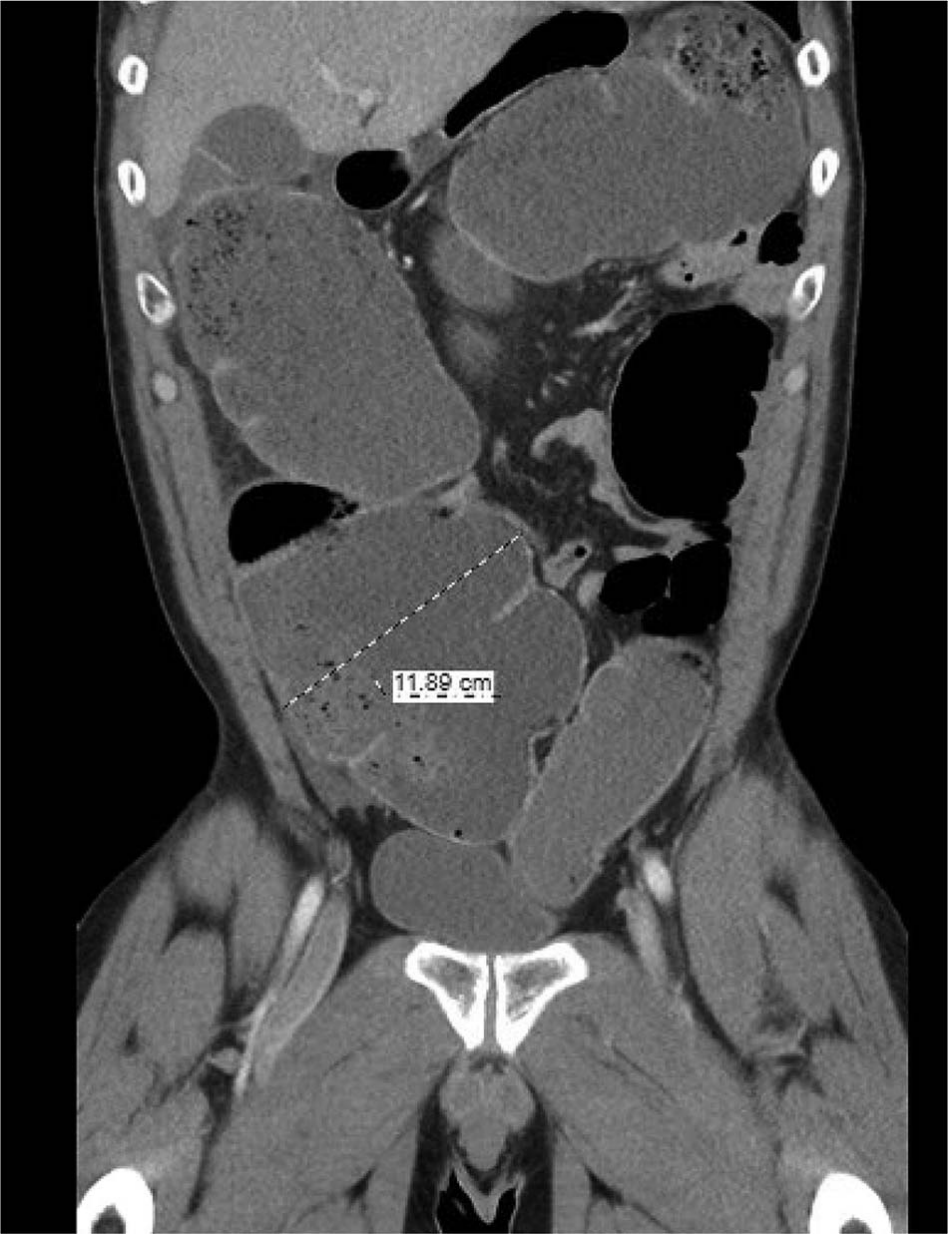
Coronal imaging demonstrating significant colonic dilation up to 11.9 cm in the cecum.

The patient was a 49-year-old male with a past medical history of anxiety and constipation who originally presented to the emergency department complaining of 1 day of worsening generalized abdominal pain, obstipation, nausea, and chills. Of note, he had undergone a colonoscopy 3 months prior to presentation, where two benign polyps were removed. There were no signs of inflammatory bowel disease. Upon examination, he was noted to be tachycardic to 130, with a blood pressure of 90/70 after 2 L of intravenous fluid resuscitation. His abdomen was distended and diffusely tender with peritoneal signs. Laboratory analysis showed a white blood cell count of 26 000, creatinine of 1.8, and a lactic acid of 7.9. A CT of the abdomen/pelvis revealed a cecum of 12 cm filled with liquid stool, but no pneumoperitoneum, pneumatosis, or colonic wall thickening. Figures 1 and 2 demonstrate the axial and coronal images of the dilated colon, respectively.

Given his acute abdomen and hemodynamic instability, the patient was urgently taken to the operating room for an exploratory laparotomy. Intraoperatively, the surgical team performed a subtotal colectomy after the cecum, ascending, descending, and majority of the sigmoid colon were all noted to be ischemic. The distal sigmoid was questionable for ischemia, but there was obvious venous congestion. Due to these findings and the patient requiring 0.2 of Levophed by that point, the decision was made to place a temporary abdominal closure device (Abthera), leave him in discontinuity, and return to the OR the following day. Tissue specimens were sent for pathological analysis.

During the second case, the rectal stump was viable. He was given an end ileostomy and returned to the ICU intubated for close hemodynamic monitoring, but no longer required vasopressor support.

He was extubated the day following his second operation. His nasogastric tube was kept in place until the ileostomy began functioning, which happened on post-operative day 3. Tissue analysis revealed ulceration and ischemic mucosal change, but no evidence of inflammatory bowel disease or *C. difficile*. The rectal stump was viable without signs of infection or ischemic necrosis. He slowly advanced to a regular diet, completed a course of broad-spectrum antibiotics, and was discharged home after a 6-day hospital course.

## Discussion

We present a novel case of TM likely induced by use of the antipsychotics aripiprazole and clomipramine. The origin was broadly considered, however no immediately antecedent event was evident from his clinical history. The most likely etiology was chronic constipation due to use of multiple antipsychotics and antidepressants. He noted no change in his overall bowel habits and reported using Metamucil as per his usual schedule.

Prior to assuming this etiology, a thorough workup including exclusion of electrolyte disturbances, thyroid hormone dysfunction, thorough medication review (particularly looking for recent antibiotic use), infectious workup evaluating for Clostridium, and colonoscopy to exclude inflammatory bowel disease must be completed [8].

Side effect profiles of antipsychotic/antidepressant medications often include constipation. Of this class, clozapine has the highest incidence of intestinal dysmotility, with multiple studies highlighting clozapine-induced gastrointestinal hypomotility [9, 10]. The mechanism by which this occurs is complex but is likely due to inhibition of the neurotransmitters serotonin, acetylcholine, and histamine, which all play roles in intestinal mobility [11]. This results in prolonged gastrointestinal transit time, leading to constipation. While studies of aripiprazole and venlafaxine did not demonstrate severe constipation like clozapine, the study did show that concomitant use of multiple antipsychotics increases the risk [12].

## Conclusion

Toxic megacolon remains a highly morbid disease process requiring prompt diagnosis and urgent intervention. Although intestinal ischemic necrosis with use of antipsychotic and antidepressant therapy is rare, this case potentially strengthens an argument to provide close clinical monitoring of patients on these regimens. Patient reports of non-specific sequelae such as constipation and vomiting may warrant further workup.
